# Antipsychotic patterns of use in patients with schizophrenia: polypharmacy versus monotherapy

**DOI:** 10.1186/s12888-014-0341-5

**Published:** 2014-11-30

**Authors:** Maxine D Fisher, Kathleen Reilly, Keith Isenberg, Kathleen F Villa

**Affiliations:** HealthCore, Inc., 800 Delaware Ave. 5th Floor Wilmington, Delaware, 19801-1366 USA; Wellpoint, St. Louis, Missouri, USA; Jazz Pharmaceuticals, Inc., 3180 Porter Drive, Palo Alto, CA 94304 USA

**Keywords:** Schizophrenia, Polypharmacy, Claims-based analysis, Patterns of use

## Abstract

**Background:**

The objective of this study was to characterize real-world treatment patterns in the prescription of antipsychotic polypharmacy (≥2 concurrent antipsychotics) compared with antipsychotic monotherapy for patients with schizophrenia.

**Methods:**

This study was a retrospective claims-based analysis of patients (aged 13–64 years) with schizophrenia belonging to an employer-based health plan. Duration of therapy was measured as the number of treatment days over one year following the initial date of antipsychotic therapy. Discontinuation was defined as a 90-day gap in antipsychotic treatment (or in at least one antipsychotic for the polypharmacy group). Logistic regression analyses were used to predict discontinuation within one year. Ordinary Least Squares (OLS) regressions were used to predict duration of therapy (by type of therapy) when controlling for gender, region, number of somatic and psychiatric comorbidities, Deyo-Charlson comorbidity score, and number of psychiatric and somatic medications.

**Results:**

Of the 4,156 patients, 3,188 received monotherapy and 968 received polypharmacy. Mean age was 40 years (37.8 years for polypharmacy vs 40.3 years for monotherapy, p < 0.001). Within one year, 77% of the polypharmacy group and 54% of the monotherapy group discontinued treatment. The average duration of therapy was 163 [SD = 143] days in the polypharmacy group vs 253 [SD = 147] days in the monotherapy group. In both cohorts, patients <25 years had a higher frequency of discontinuations than those ≥26 years. Age and polypharmacy were independent predictors of treatment duration and discontinuation prior to one year.

**Conclusions:**

One quarter of patients with schizophrenia received antipsychotic polypharmacy. Discontinuation was higher in the polypharmacy group. Age and polypharmacy were significant predictors of treatment discontinuation.

## Background

Antipsychotics have limited impact on the negative symptoms of schizophrenia and are not consistently or fully effective against positive symptoms [1,2]. This can lead to poor patient outcomes, including relapse and hospitalization [3,4]. Even with adequate antipsychotic treatment, many patients with schizophrenia continue to experience symptoms: according to the W-SOHO study, 15–39% experience a persistent symptomatic course and 14–21% of those experiencing remission subsequently relapse within a three-year period [5]. Current clinical practice commonly involves combining antipsychotics to improve treatment of patients with suboptimally controlled symptoms of schizophrenia, despite the lack of robust evidence for this approach, the increased risk of side effects, and the cost implications [6–9]. Polypharmacy prevalence rates vary widely from 2–70% [10–21] depending on study design, patient population, diagnosis, and geographical region, with a median of 19.6% of patients worldwide receiving multiple antipsychotics [22]. Polypharmacy prevalence is expected to continue to rise as more antipsychotics become available and as physicians become more familiar with the practice [11,22,23].

Evidence supporting polypharmacy is lacking, however. The majority of published studies regarding the use of two or more antipsychotics are limited to combinations with clozapine – which held the promise of improving effectiveness by taking advantage of medications with different mechanisms of action and side-effect profiles [6,8,10,24–27] – and have not demonstrated superior efficacy of polypharmacy [10,24,27–29]. Initially, it was believed that combining antipsychotics at lower doses could improve effectiveness without increasing the risk of treatment-related side effects associated with escalating doses of single agents [10]. However, polypharmacy has been associated with excessive total antipsychotic doses, which may increase dose-related side effects, the risk for drug interactions, and complicated dosing regimens that may lead to treatment discontinuation [10,16,28,29]. In the absence of clear benefit, treatment guidelines largely discourage the use of antipsychotic polypharmacy for the treatment of schizophrenia [30]. Polypharmacy is recommended for limited situations in which the patient has failed at least three trials of antipsychotic monotherapy of adequate dose and duration, including clozapine [29].

Along with the paucity of research regarding the efficacy of polypharmacy, little is known about the characteristics of patients who receive polypharmacy versus monotherapy for the treatment of schizophrenia. To fill these gaps in current knowledge, this study characterized real-world treatment patterns, including medication switching and discontinuation, in patients with schizophrenia treated with antipsychotic monotherapy versus those treated with polypharmacy.

## Methods

This retrospective cohort analysis included patients selected from the HealthCore Integrated Research Environment (HIRE), a database of medical, laboratory, and pharmacy claims of commercially insured patients. The HIRE contains administrative claims data from 14 major commercial health plans across the United States, representing 45 million lives.

As a non-interventional, observational analysis, no patients were directly involved in the study. The study was conducted in compliance with state and federal laws, including the Health Insurance Portability and Accountability Act (HIPAA) of 1996.

A chart review was included in this study and approval for a complete waiver of HIPAA authorization was granted by a central Institutional Review Board (Quorum Review IRB, waiver #26699) prior to chart collection. All claims were from a limited dataset with anonymized patient information.

### Patients

Patients between the ages of 13 and 64 years who had two or more claims for schizophrenia (International Classification of Diseases, 9th edition code 295.xx) from January 1, 2007 through to April 30, 2010 were included in the study. The younger patients were included in order to be able to describe the treatment patterns not only for chronic schizophrenia patients, but also for those who are at the earliest stages of their diagnosis and disease. Patients were followed for one year subsequent to the first claim for schizophrenia (the index date).

Antipsychotic medications were identified from pharmacy claims closest to the index date (±90 days). All first-generation (FGA) and second-generation (SGA) antipsychotics were included. Patients with a prescription for an FGA and/or SGA within 90 days before or after the index date were stratified by type of therapy into the monotherapy or polypharmacy cohorts. The first of these prescriptions was defined as the index medication. Monotherapy was defined as a single antipsychotic medication, whereas polypharmacy was defined as having two or more antipsychotics within a 45-day period. Short-acting antipsychotics given by injection for acute treatment were excluded to eliminate the possibility of mis-categorizing patients taking medications in an inpatient setting. Long-acting injections were not included because we found their use to be low in the claims (<2% overall, data not shown).

From the list of all schizophrenia patients included in the study, a random sample of 200 was targeted for medical record review. Patients were required to have documented schizophrenia in their medical record.

### Outcome measures

Because clinical outcome measures are not available in administrative claims data, the primary outcome measure used in this analysis was duration of therapy, measured over one year following the date antipsychotic treatment was initiated. Treatment duration was defined as the number of days without a 90-day gap in treatment. Discontinuation was defined as a 90-day gap in antipsychotic therapy prior to the end of the one year follow-up period. In the polypharmacy group, discontinuation was defined as a 90-day gap in at least one of the antipsychotic medications during follow-up.

Secondary outcomes included the type and number of psychiatric and somatic comorbidities during the one year follow-up period. Psychiatric comorbidities included depression, anxiety, bipolar, schizoaffective, attention-deficit and personality disorders. Analysis of somatic comorbidities included the Deyo-Charlson Comorbidity Index (DCI) score [31] and the proportion of patients with claims for cardiovascular diseases, cerebrovascular disease, diabetes, hyperglycemia, or hyperlipidemia. Concomitant psychiatric medications other than antipsychotics, including antidepressants, anxiolytics, hypnotics and sedatives, mood stabilizers, and stimulants, were recorded. The type and number of somatic medications, including lipid-modifying agents, anti-diabetic agents, anti-Parkinson disease and movement disorder medications, cardiovascular agents, and anti-obesity drugs were also collected.

A random sample of 200 patients within the larger claims cohort was selected for medical chart review to estimate schizophrenia severity because this is not available in claims data. Severity within the sub-sample was determined from physician documentation in the charts and described as mild, moderate or severe. Due to the small number of patients with documented schizophrenia severity, comparisons between polypharmacy and monotherapy groups were descriptive only.

### Statistical analysis

Measures were described as means (± standard deviation [SD]) for continuous variables, and frequencies for categorical variables. Differences in mean length of therapy were compared using *t* tests, and χ^2^ tests were used to compare the proportion of patients discontinuing treatment and the prevalence of comorbidities. Ordinary Least Squares (OLS) regression was used to predict the length of therapy (by type of antipsychotic therapy regimen), when controlling for gender, region, number of somatic and psychiatric comorbidities, DCI score, and the number of psychiatric and somatic medications.

## Results

### Patient characteristics

A total of 4,156 patients were included in the study. Of these, 3,188 were in the monotherapy cohort and 968 were in the polypharmacy cohort (Table 1). Overall, 53.1% were male (53.6% in the monotherapy group and 51.2% in the polypharmacy group).The mean age was 40 years, with patients in the polypharmacy group significantly younger than those in the monotherapy group (37.8 vs. 40.3 years, respectively, p < 0.001).

**Table 1:** **Patient characteristics at baseline (6 months pre-index)**

**Characteristic**	**Total (N =4,156)**	**Monotherapy (n =3,188)**	**Polypharmacy (n =968)**	**p-value** ^**a**^
Length of follow-up, days (mean, SD)	993.5 (391.8)	989.3 (393.8)	999.3 (397.1)	.488
Gender, n (%)				
Male	2206 (53.1)	1,710 (53.6)	496 (51.2)	.190
Age at index date, years (mean, SD)	40 (13.7)	40.3 (13.6)	37.8 (13.6)	<.001
Psychiatric comorbidities, n (%)				
Depression	1090 (26.2)	806 (25.3)	284 (29.3)	.012
Schizoaffective disorders	991 (23.8)	786 (24.7)	205 (21.2)	.026
Anxiety disorders	441 (10.6)	336 (10.5)	105 (10.8)	.786
Bipolar disorder	247 (5.9)	190 (6.0)	57 (5.9)	.934
Personality disorders	128 (3.1)	94 (2.9)	34 (3.5)	.374
Attention deficit disorder	114 (2.7)	83 (2.6)	31 (3.2)	.318
Substance use disorder	83 (2.0)	59 (1.9)	24 (2.5)	.221
Psychiatric medications, n (%)				
Antidepressants	2016 (48.5)	1516 (47.6)	500 (51.7)	.025
Sedatives or hypnotics	1623 (39.1)	1051 (33.0)	572 (40.9)	<.001
Mood stabilizers	1073 (25.8)	794 (24.9)	279 (28.8)	.015
Anti-anxiety medications	926 (22.3)	678 (21.3)	248 (25.6)	.004
Stimulants and ADHD medications	40 (1.0)	32 (1.0)	8 (0.8)	.621
Mean (SD) Deyo-Charlson Comorbidity Index Score (range 0–16)	0.6 (1.0)	0.6 (1.0)	0.6 (0.9)	.546
Somatic medications, n (%)				
Anti-hyperlipidemic agents	623 (15.0)	490 (15.4)	133 (13.7)	.213
Anti-hypertensive agents	502 (12.1)	408 (12.8)	94 (9.7)	.010
Anti-thyroid agents	418 (10.1)	310 (9.7)	108 (11.2)	.194
Anti-diabetic agents	358 (8.6)	275 (8.6)	83 (8.6)	.960
Anti-Parkinson agents	60 (1.4)	37 (1.2)	23 (2.4)	.005
Random sample of patients with baseline disease severity data available from charts (n = 121), n (%)				
Mild	25 (20.7)	22 (23.2)	3 (11.5)	
Moderate	31 (25.6)	25 (26.3)	6 (23.1)	
Severe	65 (53.7)	48 (50.5)	17 (65.5)	

ADHD = attention deficit hyperactivity disorder; SD = standard deviation.

^a^Polypharmacy vs monotherapy.

At baseline, 26.2% of the overall patient population had comorbid depression (29.3% and 25.3% in the polypharmacy and monotherapy cohorts, respectively; p = 0.012) and 23.8% had comorbid schizoaffective disorders (21.2% and 24.7%, respectively; p = 0.026). There was no statistical difference in the DCI score between the two groups at baseline. Patients in the polypharmacy group had significantly higher use of concomitant psychiatric medications (other than antipsychotics) at baseline compared with the monotherapy group, notably antidepressants (51.7 vs. 47.6%), sedatives (40.9 vs. 33.0%), mood stabilizers excluding antipsychotics [27,32,33] (28.8 vs. 24.9%), and anxiolytics (25.6 vs. 21.3%). The monotherapy group had a statistically higher proportion of patients with claims for anti-hypertensive agents compared with the polypharmacy group (12.8 vs. 9.7%; p = 0.010). Claims for anti-Parkinson agents were higher in the polypharmacy group compared to the monotherapy group (2.4 vs. 1.2%; p = 0.005) (Table 1).

In the sample of 200 patient charts selected for assessment of schizophrenia severity, 188 patients were receiving antipsychotic medications. Of these 188 patients, 121 patients had a severity rating within 30 days of the index date. Among those, a higher proportion of patients in the polypharmacy cohort were rated as having severe disease compared with those in the monotherapy cohort (65.5 vs. 50.5%), whereas, more patients in the monotherapy group were rated as having mild disease than those in the polypharmacy group (23.2 vs. 11.5%) (Table 1).

### Index antipsychotic use

Table 2 outlines the specific antipsychotics and antipsychotic combinations that were used in the two groups. For the 76.7% of patients who were on monotherapy, the most frequently used antipsychotic was risperidone (24%). Among the 23.3% of patients in the polypharmacy cohort, 63.2% of the combinations included olanzapine, risperidone, or another SGA other than clozapine. The most commonly used combination was quetiapine plus risperidone (9.9%). Only 10.2% of the polypharmacy combinations included clozapine. Slightly more than a quarter of the polypharmacy combinations (26.5%) included an FGA. In comparison, only 13.4% of those in the monotherapy cohort received an FGA as index therapy.

**Table 2:** **Index antipsychotic therapies**

**Therapy**	**Total N = 4,156 n (%)**
Patients on monotherapy (% of total)	3,188 (76.7)
Number of patients by type of antipsychotic monotherapy (% of monotherapy)	
Risperidone	765 (24.0)
Aripiprazole	536 (16.8)
Olanzapine	505 (15.8)
Quetiapine	437 (13.7)
Ziprasidone	261 (8.2)
Clozapine	200 (6.3)
Other second-generation antipsychotic	57 (1.8)
First-generation antipsychotic	427 (13.4)
Total on polypharmacy (% of total)	968 (23.3)
Number of patients by type of antipsychotic polypharmacy combinations (% of polypharmacy)	
Combinations with first-generation antipsychotics	257 (26.5)
Combinations with olanzapine	216 (22.3)
Combinations with risperidone	210 (21.7)
Combinations with other second-generation antipsychotics	186 (19.2)
Combinations with clozapine	99 (10.2)
Top 5 polypharmacy combinations	
Quetiapine and risperidone	96 (9.9)
Quetiapine and aripiprazole	94 (9.7)
Quetiapine and first-generation antipsychotic	83 (8.6)
Aripiprazole and risperidone	65 (6.7)
Aripiprazole and olanzapine	65 (6.7)

### Discontinuation and duration of therapy

**Figure 1:**
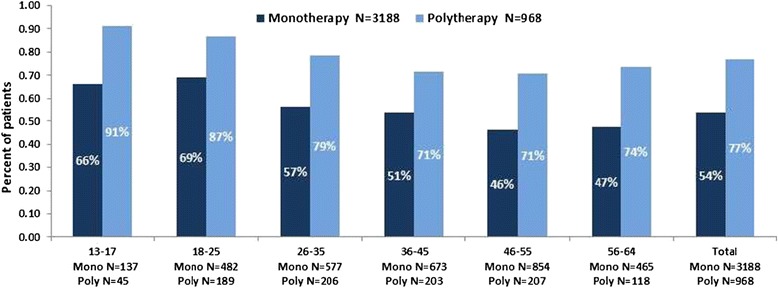
**Percentage of patients, by age, who discontinued** ^**a**^ **within one year follow-up.** ^a^For polypharmacy patients, discontinuation was defined as a 90-day treatment gap in at least 1 index antipsychotic.

A higher proportion of patients in the polypharmacy cohort discontinued index treatment prior to the end of the one year follow-up period compared with the monotherapy group (Figure 1). Overall, 77% of those in the polypharmacy group discontinued at least one of their index antipsychotics within one year, whereas 54% of those in the monotherapy group discontinued their index medication within one year. A greater proportion of patients in the polypharmacy group discontinued therapy compared with those receiving monotherapy when all age groups were considered. In both the monotherapy and polypharmacy cohorts, patients younger than 25 years had a higher frequency of treatment discontinuation prior to one year than those 26 years and older (Figure 1).

The average duration of therapy (i.e., without a gap of 90 days or more) was significantly greater for patients in the monotherapy cohort than those in the polypharmacy cohort (252.7 [SD = 147.4] vs 163.8 [SD = 143.4] days; p < 0.01; Data not shown).

Among the patients included in the chart review sample, those with greater disease severity had a higher frequency of treatment discontinuation within one year compared with patients who had mild or moderately severe disease (68 vs. 46%; Data not shown).

In multiple regression analyses, both age and use of polypharmacy were independent predictors of length of therapy defined as the number of days on the index medication (Table 3). Additionally, receiving two or more psychiatric medications (that were not antipsychotics) was also independently associated with a shorter duration of antipsychotic therapy (−13 days; p < 0.047).

**Table 3:** **Ordinary least squares (OLS) regression predicting length of therapy**

		**95% Confidence interval**		
**Covariate** ^**a**^	**Coef.**	**(lower – upper)**		**p-Value**
Polypharmacy (reference = monotherapy)	−106.638	(−117.179 – −96.096)		<.001
Female (reference = male)	−1.700	(−10.914 – 7.514)		.718
Age	1.582	(1.198 – 1.965)		<.001
Number of post-index psychiatric comorbidities of interest (reference = 0)				
One	−9.402	(−19.946 – 1.142)		.081
Two	−38.278	(−52.526 – −24.031)		<.001
Three or more	−67.391	(−87.620 – −47.163)		<.001
Number of psychiatric medications post-index (reference = 0)				
One	−4.376	(−17.546 – 8.794)		.515
Two or more	−12.682	(−25.211 – −0.153)		.047
Number of somatic medications post-index (reference = 0)				
One	20.108	(8.237 – 31.980)		.001
Two or more	13.279	(−1.724 – 28.283)		.083
Constant	218.211	(197.930 – 238.492)		<.001
*R* ^*2*^	0.148			

^a^Covariates not shown include plan region, number of somatic comorbidities, and Deyo-Charlson comorbidity index score.

**Figure 2:**
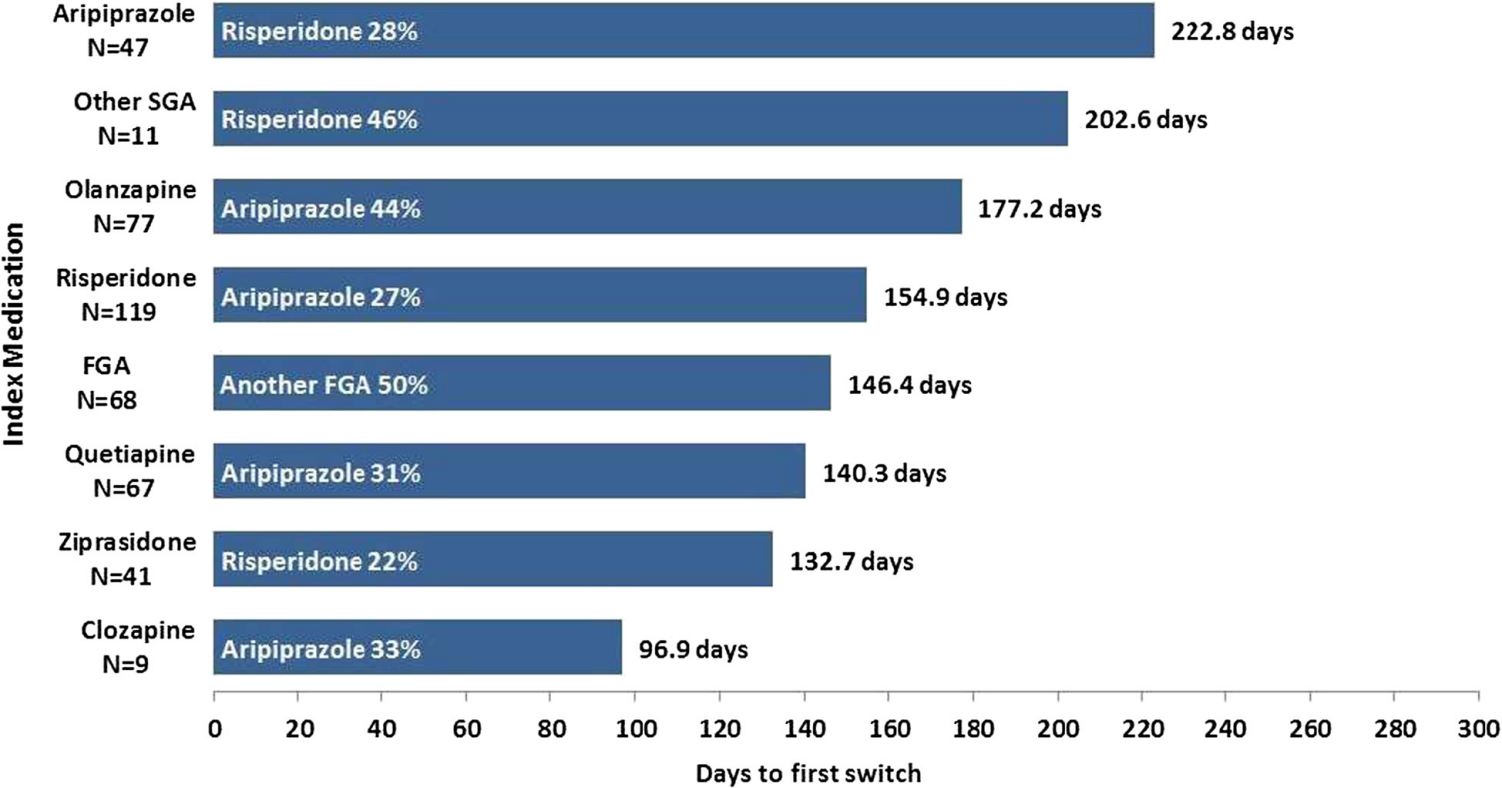
**Days to first switch by index medication by medication of most frequent switch among 439 monotherapy patients who switched antipsychotic therapy during 12-month follow-up.** FGA = first-generation antipsychotic; SGA = second-generation antipsychotic.

After discontinuing antipsychotic therapy, 10.3% of patients in the monotherapy cohort restarted their index medication during the follow-up period, while 13.8% switched to a different antipsychotic (Table 4). Commensurate with its use as a treatment reserved for patients who have failed to respond to other antipsychotics, clozapine had the lowest proportion of patients (4.5%) switching medications. Among the more commonly used antipsychotics, the patients who switched least often were those taking aripiprazole (8.8%) (Table 5). In addition, aripiprazole was the most common second medication among patients in the monotherapy cohort who switched treatment (Figure 2). Patients receiving an SGA as index therapy but later switched tended to receive a different SGA as second-line therapy. Among patients who received an FGA as the index antipsychotic and later switched, half (50%) switched to another FGA.

**Table 4:** **Antipsychotic medication status after discontinuation among patients on monotherapy who discontinued (90-day gap in therapy) during one year follow-up**

	**Total (n = 3,188)**
Patients who discontinued prior to 12 months, n (%)	1706 (53.5)
Patients who restarted medication after 90 day gap in treatment, n (%)	317 (10.3)
Mean time to restart, days (SD)	182.7 (59.3)
Patients who switched medications, n (%)	439 (13.8)
Average time to first switch, days (mean, SD)	166.9 (100.7)
Number of switches, n (%)	
One	356 (81.1)
Two	58 (13.2)
Three or more	25 (5.7)

**Table 5:** **Overall rate of switch during one year follow-up by index monotherapy**

**Index therapy**	**Patients switching from index therapy n (%)**
Aripiprazole	47 (8.8)
Clozapine	9 (4.5)
Olanzapine	77 (15.2)
Quetiapine	67 (15.3)
Risperidone	119 (15.6)
Ziprasidone	41 (15.7)
Other second-generation antipsychotic	11 (19.3)
First-generation antipsychotic	68 (15.9)
**Total**	**439 (13.8)**

Among patients in the polypharmacy cohort who discontinued at least one of their index antipsychotics, 39.1% switched to monotherapy. Of those who switched to monotherapy, 21.1% remained on one of their index antipsychotics while 18.0% switched to a different agent (Table 6). Of the 17.2% of patients who remained on a polypharmacy regimen, 8.9% received a combination that included one of their index medications; 8.3% were switched to a polypharmacy regimen that included different medications. A substantial proportion of patients in the polypharmacy group who discontinued index therapy did not receive antipsychotics (43.8%) for the remainder of the follow-up period.

**Table 6:** **Antipsychotic switching among patients receiving polypharmacy who discontinued (90 day gap in therapy) ≥1 index antipsychotics during one year follow-up**

	**n (%)**
Patients receiving polypharmacy^a^ who discontinued ≥1 index medications	745 (77.0)
Antipsychotic medication status after discontinuation	
Monotherapy with 1 of the index medications	157 (21.1)
Monotherapy with switch to a new antipsychotic	134 (18.0)
New polypharmacy regimen including 1 of the index medications	66 (8.9)
New polypharmacy regimen with all new antipsychotics	62 (8.3)
Remained on no antipsychotics	326 (43.8)

^a^n = 968.

## Discussion

In this real-world cohort analysis, 23% of patients with schizophrenia were prescribed two or more antipsychotic medications. Although prevalence rates in previous studies ranged from 2% to 70% [10–21], our findings are consistent with a worldwide median of 19.6% [21,22]. It is expected that the rates reported in our claims analysis would be lower than those reported elsewhere given that the database consisted of patients with schizophrenia who were members of employer-based health plans. Such patients would be expected to have lower schizophrenia severity as they would be required to maintain either steady employment or family relationships to retain health plan eligibility.

We found the highest polypharmacy rates among patients with more severe schizophrenia. Similarly, medical chart review suggested that more severe schizophrenia was seen in patients receiving polypharmacy. Among the youngest patients, whose early course may signal greater schizophrenia severity, polypharmacy rates were higher than those seen in older patients. It is possible that some patients using short-term polypharmacy were mis-classified in our study as receiving polypharmacy, causing slightly higher percentages than those in other studies. None of the patients categorized as polypharmacy had fewer than two concomitant fills. Injectable medications were excluded.

While approximately a quarter of patients receiving polypharmacy were prescribed a combination that included an FGA, the most frequently used combinations across all classes were combinations of SGAs. An additional examination of the treatment patterns by age showed that combinations of two FGAs were more frequent among patients in the two oldest age groups, whereas combinations with SGAs were more common in the younger age groups. Furthermore, our data show that patients who were transitioned off monotherapy were more often prescribed a second antipsychotic to the one that they had failed, rather than started on two new medications. Thus, within the older age groups, treatment patterns were consistent with guidelines that recommend polypharmacy only after the patient has failed trials of at least one additional SGA, an FGA, and finally clozapine. However, in our study population, the use of clozapine either alone or in combination was low.

Treatment patterns may have differed among age groups based on timing and availability of newer antipsychotics. Still, these data suggest that many physicians did not wait for patients in younger age groups to fail multiple types of treatments before moving to an antipsychotic polypharmacy regimen. This finding is consistent with previous research showing that physicians tend to prescribe multiple antipsychotics before exploring the full dose range of several different single antipsychotic agents [10,19,33]. Although it is understandable that physicians want to control severe acute symptoms of schizophrenia, particularly early in the course of disease, long-term use of polypharmacy beginning at an early age may pose additional risk to patients and warrants further study. Physicians may perceive that use of two SGAs is safer than switching a patient to an SGA, then to clozapine, but these attitudes may be changing with the growing body of evidence that SGAs carry their own serious adverse effects on physical health [34–36]. The use of concomitant psychiatric medications was not only high but also higher among patients in the polypharmacy cohort than the monotherapy cohort. The high use of benzodiazepines is of concern given the recent findings of morbidity and mortality links to benzodiazepine use and polypharmacy use among patients with schizophrenia [10].

The discontinuation rate of 77% among patients in the polypharmacy cohort is high, but consistent with prior research [34]. The shorter duration of therapy in the polypharmacy cohort may have been related to concerns by physicians over the long-term use of two antipsychotics and may have been an intentional strategy. It is difficult to tease apart non-adherence/persistence on the part of patients from prescribing decisions made by physicians in claims. Another limitation of claims analyses is that we are unable to estimate optimum antipsychotic dose. The post-medication status of patients who entered the study on polypharmacy suggests a number of possible circumstances. In keeping with the known challenges of adherence among patients with schizophrenia, combinations of antipsychotics, which require more complex dosing regimens, and the overall higher pill burden, may make adherence among these patients all the more difficult [10]. Furthermore, lack of efficacy and emergence of side effects may also have led treating physicians to abandon the strategy of polypharmacy. Unfortunately, for patients with severe symptoms that do not fully remit with current treatments, few alternatives are yet available.

## Conclusions

In a real-world analysis, 23% of patients with schizophrenia received treatment with two or more antipsychotic agents simultaneously. Discontinuation rates were higher among patients receiving polypharmacy compared with those receiving monotherapy. Patient age and use of polypharmacy were independent predictors of length of therapy over one year of follow-up.
